# Feasibility and effectiveness of a smartphone access program for promoting engagement in care among perinatal people with substance use disorders: a pilot study

**DOI:** 10.3389/frhs.2025.1640311

**Published:** 2025-09-12

**Authors:** D. J. Goodman, L. Lamadriz, K. Stokes, M. Adams, H. Martell, K. Robie, A. Morgan, E. C. Saunders

**Affiliations:** ^1^ Geisel School of Medicine, The Dartmouth Institute, Hanover, NH, United States; ^2^ Department of Obstetrics and Gynecology, Dartmouth-Hitchcock Medical Center, Lebanon, NH, United States; ^3^ Center for Technology and Behavioral Health, Geisel School of Medicine at Dartmouth, Hanover, NH, United States

**Keywords:** perinatal care, feasibility, implementation, engagement, social needs, healthcare disparities, substance use disorder, smartphones

## Abstract

**Introduction:**

Pregnant and postpartum people with substance use disorders (SUD) experience high rates of morbidity and mortality, especially postpartum. For this vulnerable group, lack of access to a phone contributes to poor engagement in perinatal care. This paper describes initial work evaluating the implementation of a free smartphone program for rural pregnant patients with SUD and its effectiveness for improving participation in care.

**Methods:**

This retrospective type I hybrid-effectiveness cohort study evaluated program effectiveness, acceptability, and feasibility of implementation in obstetric practice. Semi-structured interviews with patients, providers and obstetric staff (*n* = 8) explored implementation success. Data on phone utilization, engagement in care and outcomes were abstracted from electronic health records and compared among three cohorts (Cohort 1: patients with SUD who received phones; Cohort 2: patients with SUD not receiving phones; Cohort 3: Patients without SUD). Kruskal–Wallis and chi-squared/Fisher's Exact tests were utilized for comparisons.

**Results:**

Providers, staff, and patients universally found the smartphone access program useful, perceiving that it improved patient engagement in digital and in-clinic care. From 2021 to 2024, 44 patients with SUD participated in the smartphone program for an average of 162 days. Cohort 1 entered prenatal care later, attended fewer prenatal visits, and were more likely to have psychiatric comorbidity than Cohorts 2 and 3. After receiving a smartphone, there were no differences in postpartum visits between cohorts, and higher rates of behavioral health and recovery support for Cohort 1.

**Discussion:**

In a rural obstetric clinic, implementing a free smartphone program for perinatal patients with SUD was feasible and acceptable. Though there was no difference in prenatal care utilization, patients who received a smartphone engaged in robust postpartum care and behavioral healthcare utilization. Addressing digital disparities is an essential component of health equity.

## Introduction

Over the past two decades, substance use has become a leading cause of morbidity and mortality for pregnant and postpartum people (1–8), accounting for an estimated quarter of maternal deaths during the perinatal period (7). Engagement in perinatal care and SUD treatment is associated with improved outcomes for birthing persons and their infants (9–11), including reduced risk for preterm delivery (11) and increased infant birthweight (11, 12). Despite the importance of participating in perinatal care, rates of prenatal care engagement are low for pregnant people with SUD, with one recent study estimating that only 50% of birthing persons with SUD in the United States (US) receive adequate prenatal care (13). These healthcare disparities also extend to the postpartum period, where birthing persons with SUD have a 53% decrease in the odds of attending a postpartum visit with an obstetric provider (14).

To reduce overdose mortality during pregnancy and postpartum, mitigating environmental and resource disparities is crucial. According to the Health Equity Framework for Maternal Mortality by Kramer and colleagues (15), addressing only individual-level biomedical and behavioral causes of maternal death is inadequate because of population-level differences in drivers of morality rates. The socio-contextual environment, including social determinants of health (SDOH), acts as a mortality risk regulator, constraining and influencing individual behaviors and impacting health care access (15). Therefore, community-level interventions addressing these SDOH disparities, including digital determinants of health (16), could be transformative by supporting access to health care.

As technology increasingly becomes implemented into routine health care, lack of access to digital technology, including smartphones, can reinforce health disparities (16) and could actually worsen gaps in access to care (17–19). Digital health tools that facilitate telehealth and digital engagement with healthcare providers are a vital resource for pregnant and postpartum patients (20–22). In addition to virtual visits, digital platforms allow patients to communicate asynchronously with providers through patient portals, complete mental health and other medical screenings, check lab results, view providers instructions, engage with community health workers for social support, schedule and cancel appointments and arrange transportation. However, with rapid transition to virtual engagement with healthcare over the past decade, new disparities in healthcare access have become apparent. Digital disparity in phone access may be especially concerning for rural pregnant and postpartum persons. While one small cross-sectional survey estimated that 88% of pregnant women with SUD in urban and suburban regions had access to a mobile phone or smartphone (23), another study found that the majority of rural obstetric patients with SUD had never owned or used a smartphone (24). Patients with SUD often share phones, presenting an additional barrier to engaging with healthcare providers when needed, and possible privacy concerns (20). Without adequate phone or internet access to schedule appointments, receive reminders, or participate in telehealth, low-income patients often struggle to attend scheduled visits and remain engaged in care (25). This results in missed appointments, inability to communicate with providers, and a worsening of existing disparities in access and outcomes for rural patients with the most limited resources (24, 26). As described by the Kramer Health Equity Framework for Maternal Mortality (15), community-level interventions providing access to phones could be a facilitator of access to health care by enhancing resources for pregnant and postpartum patients (15).

Research exploring solutions to the challenges of providing equitable access to telehealth for patients with limited resources is sparse, although one study suggests that providing rural obstetric patients with a free smartphone and data plan has the potential to improve access and participation in obstetric care (24), and another found that providing phones may empower pregnant people to seek health services (27). Several studies have examined the impact of providing mobile phones to obstetric patients in low resource settings, including primary care and obstetrics clinics serving low income pregnant and postpartum people in the United States (24) and Nigeria (28), suggesting that providing phones may contribute to increased overall healthcare engagement (24, 28) as well as expanded access to web-based postpartum health information (19). Despite these potential benefits, we were unable to identify any studies which provided smartphones to obstetric patients with SUD, or which examined determinants influencing the implementation of a free smartphone program. This manuscript describes a pilot program to provide free smartphones to obstetric patients with SUD, and provides preliminary data exploring the implementation of the program, the characteristics of patients engaged in the program, and the prenatal and postpartum care utilization of patients engaged in the program.

Smartphone access was identified as a significant barrier to engagement in prenatal care for pregnant persons with SUD at Dartmouth Hitchcock Medical Center (DHMC). DHMC is located in rural central New Hampshire (NH) and serves rural and remote rural areas of NH and Vermont (VT), in addition to accepting regional referrals for high-risk obstetrics. Surrounding communities have high rates of substance use overdose deaths (29), consistent with many rural areas of the US. Drug overdose is the leading cause of maternal mortality in NH, with the majority of deaths occurring during the postpartum period. Improving participation in prenatal and postpartum care to reduce drug overdose mortality is therefore an important goal at DHMC. Historically, only 40% of patients with SUD receiving prenatal care or delivery services at DHMC participate in postpartum care, less than half the rate of DHMC obstetric patients overall. This disparity in care reflects the significant barriers faced by rural people with SUD, often including lack of access to phones, broadband internet, or stable housing. During the first phase of the COVID pandemic, surveys of obstetric patients at DHMC identified barriers to telehealth access, including poor broadband internet connectivity in rural and remote rural areas, the cost of internet and/or mobile phone plans, lack of access to a mobile phone, or inadequate data plans precluding engagement in telehealth (30). For example, a smartphone user with a limited budget plan may expend most cell phone minutes allocated for an entire month in a 30-minute telehealth visit, leading to data overage charges or loss of service (30). These challenges disproportionately impacted pregnant patients with SUD, who typically had very limited resources.

To address disparities in digital access and facilitate access to perinatal care for pregnant and postpartum people with SUD, the Department of Obstetrics and Gynecology (OB/GYN) at DHMC developed a novel program to provide free smartphones and unlimited calling, texting and data plans to improve perinatal care engagement for this population, facilitated by community health workers and a recovery support workers (RSW) embedded in the obstetric outpatient clinic. Qualitative data obtained in initial evaluation of the cell phone program indicated popularity with patients, and strong endorsement from clinical providers, but did not examine implementation characteristic or impact in terms of healthcare utilization or clinical outcomes.

### Study aims

To understand the implementation process and impact of the smartphone access program, we conducted semi-structured qualitative interviews with patients and providers on the impact of the program. To examine the initial effectiveness of this program, a single site, retrospective cohort study was conducted to examine smartphone utilization and rates of perinatal care engagement, including telehealth engagement in addition to feasibility of implementation. To explore rates of prenatal and postpartum engagement, we compared telehealth and clinic-based service utilization among three cohorts of birthing people, including prenatal and postpartum patients with SUD enrolled in the smartphone access program (Cohort 1), patients with SUD not enrolled in the smartphone access program (Cohort 2), and patients without SUD not enrolled in the smartphone access program (Cohort 3). We hypothesized that providing free smartphone access for low-resource, rural pregnant patients would improve engagement in digital and in-person prenatal and postpartum care services and reduce SDOH-driven disparities in prenatal and postpartum care attendance.

The aims of the manuscript are, 1) to describe the implementation success (adoption, implementation, and sustainment) and impact of the smartphone access program, as defined in the updated Consolidated Framework for Implementation Research (CFIR) (31) and 2) to examine the preliminary effectiveness of the free smartphone program for increasing engagement with prenatal and postpartum care, an important clinical outcome. Future qualitative analysis will provide a more in-depth discussion of the process of embedding a free smartphone program into existing clinical workflows in a busy rural obstetric practice, implementation barriers and facilitators, and the intersecting relationships between implementation and structural power imbalances which impact engagement in healthcare for this target population.

## Methods

### Study design

This retrospective cohort study utilized a type I hybrid-effectiveness study design (32) to gather preliminary data on program effectiveness, while examining the feasibility of implementing the smartphone access program into the clinical workflow. A type I hybrid-effectiveness study has the primary aim of assessing the impact of a clinical intervention on clinical outcomes, but also collects data on the implementation of the intervention (33). We focused on assessing the success (rather than the process) and impact of the smartphone access program's *implementation,* by conducting qualitative semi-structured interviews with patients, clinicians, community health workers, and administrators. Program *effectiveness*, including phone utilization data, were abstracted from the electronic health records (EHR) of patients with SUD enrolled in the smartphone access program, patients with similar social determinant of health (SDOH) needs who did not receive a smartphone or data plan, and commercially insured patients without SUD, who reflected most of the patients seen by the practice. This study was funded by an institutional research grant and approved by the Dartmouth Health Institutional Review Board.

### Origins of the smartphone access program

During the beginning of the COVID-19 pandemic, when obstetric patients were being advised to avoid physical contact with health systems and the majority of care was abruptly moved to telehealth, the OB/GYN department at the rural medical center initiated a program to repurpose donated smartphones and computers which were distributed to pregnant and postpartum patients who lacked access to digital technology. Based on patient feedback regarding barriers to accessing digital healthcare (30) and the experience of this pandemic inspired innovation, OB/GYN subsequently launched a free smartphone program for pregnant patients with SUD, supported initially by institutional funding. Patients with SUD who lacked access to a phone were eligible to receive a pre-paid smartphone and renewable talk, text and data cards, enabling participation in both virtual provider visits as well as other forms of digital engagement with healthcare and recovery support. After the first two months of the program, data cards were upgraded to unlimited talk, text, and data, in response to patient and provider feedback that a limited data plan was insufficient to support access to telehealth video visits, a data-hungry modality. To obtain a phone, patients met with a recovery support worker (RSW) dually trained as a community health worker embedded in the OB/GYN department, to help with phone set-up and provide technical assistance to enable patients to access their own electronic health records. Prepaid smartphones do not require a contract and were purchased by a member of the clinical team from a local retail chain, with data packages uploaded electronically each month by texting a link to the phone recipient.

Phone numbers for the OB/GYN clinic, the RSWs, the Medicaid transportation scheduling line, and other regional resources were programmed into the phones. Phones and data were renewed monthly by the RSWs during pregnancy and initially through 3 months postpartum, program support was subsequently extended through one year postpartum with the receipt of additional funding. The RSWs tracked phone deployments and data packages on an excel spreadsheet in a secure drive behind the institutional firewall to maintain patient privacy. Using the smartphones, patients were able to access their electronic health records, schedule visits virtually or by telephone, engage in telehealth, and send and receive messages from their providers. Patients also received synchronous and asynchronous support from the RSWs by text messaging, phone, or in-person. Participants could interact with the RSWs as needed during pregnancy and postpartum for assistance connecting with services, and support during prenatal care visits and the delivery hospitalization. At the end of their period of support by the phone program, participants were able to keep their cell phones and purchase their own data plans.

### Study participants

Obstetric patients, clinicians, community health workers and department administrators were recruited to participate in semi-structured interviews. Patients who received a cell phone were recruited through invitation by members of the OB team (clinicians, community health workers), staff and administrators who were involved in implementing the program and/or referring patients for phones were invited by email from the PI (DG) or study team member AM. Staff and administrator interviews were conducted shortly after implementation to ensure recall of implementation details; patient interviews were conducted between 2021 and 2025.

Participants in the retrospective cohort arm of this mixed methods study were pregnant or postpartum patients enrolled in obstetric care at DHMC who delivered between September 1, 2021, and December 31, 2024. Patients were eligible for enrollment in the smartphone access program if they enrolled in obstetric care, gave birth or were transferred to DHMC at the time of delivery, had a SUD diagnosis documented by ICD-10 code, and self-reported lack of access to a smartphone or adequate data plan. We compared prenatal and postpartum healthcare utilization and perinatal outcomes for this cohort to those of obstetric patients with or without SUD who were not enrolled in the phone access program. In total, three cohorts of pregnant and postpartum patients were included, matched by year of delivery (2021–2024): (1) Cohort 1: Patients with SUD enrolled in the smartphone access program; (2) Cohort 2: Patients with SUD not enrolled in the smartphone access program, and (3) Cohort 3: Patients without SUD not enrolled in the smartphone access program. Historical data from the DHMC obstetric program shows that patients with SUD have significantly less utilization of prenatal and postpartum care. Our work is based in the premise described by Kramer and colleagues (15), that fundamental social determinants (such as disparities in access to digital technology) which limit participation in clinical care, heighten risks for morbidity and mortality. Pregnant patients with SUD who lacked access to a smartphone are among those with the most severe social and economic challenges in our patient population. To understand the potential of the smartphone program to begin to narrow the gap in access, we were interested in comparing utilization of obstetric care for patients who received a smartphone with those who already had access to one. We included Cohort 2, as this cohort of patients with SUD would likely have similar SDOH vulnerabilities. Cohort 3 was included to provide a comparison group without SUD whose engagement in care would reflect the experience of those patients with the “usual” SDOH vulnerabilities.

Patients in Cohorts 2 and 3 were identified through random selection. The Dartmouth Analytics Core generated a random list of patients filtered by dates of delivery admission. Charts selected for Cohort 2 were also filtered to include patients with documented SUD (ICD10 codes for SUD), while charts selected for Cohort 3 had no documented SUD. Patients in Cohorts 2 and 3 were only matched on to those in the smartphone access group (Cohort 1) by age and year of delivery. Patients in Cohorts 2 and 3 were screened for phone access. During the abstraction process, the Research Nurse also confirmed that participants in Cohorts 2 and 3 had documented access to a phone, as all patients were screened for stable phone access during their prenatal care. Because this was a small pilot project, no sample size calculations were conducted. All patients enrolled in the smartphone access program (Cohort 1; *n* = 44) were enrolled in the study. Sample sizes for Cohorts 2 (*n* = 43) and 3 (*n* = 44) were determined by the number of participants enrolled in Cohort 1, so the cohorts were matched in size.

### Data collection and procedures

Qualitative data were collected through semi-structured interviews with perinatal clinic staff and patients. All interviews were conducted by telephone by AM. Interviews were guided by a semi-structured interview guide developed by the study team, which included people with lived experience of SUD, and informed by the domains of the Consolidated Framework for Implementation Research CFIR (34). Interviews were approximately 30 min in length and were transcribed by members of the study team.

Data on phone deployment were collected by the recovery support workers for all patients enrolled in the smartphone access program. Demographics, utilization of obstetric care, and perinatal outcomes data were abstracted for all cohorts from EHRs by a trained Research Nurse (LL). De-identified data were entered into DHMC's REDCap (Research Electronic Data Capture) system, a secure, web-based application designed to support data capture for research studies (35). Data collection procedures were piloted for several records by LL, a skilled Research Nurse with over 20 years of experience in obstetrics and five years in obstetric record abstraction, then verified for accuracy by the study's clinical lead (DG). No disagreements were found between the two. Data was then abstracted from each record and recorded in REDCap. Any fields where interpretation was unceratin were discussed with the second reviewer (DG) and consensus achieved, DG and LL also met weekly to discuss any questions about interpretation of the data elements.

### Outcome measures

The impact of the free smartphone program was examined through qualitative interviews with patients, and clinical staff focused around implementation and impact as defined below in the CFIR. Additionally, the implementation effectiveness was explored through quantitative phone utilization, including the number of cell phones distributed and days of phone use that were supported by the program; the gestational age at the time of phone deployment, and whether a phone had to be replaced.

Effectiveness data were abstracted from patient's obstetric treatment records. The primary outcomes of the study were postpartum engagement and preterm birth.

− The number of cell phones distributed;
− The number of days of use that were supported by the program;
− The gestational age at the time of phone deployment;
− Whether a phone had to be replaced.

Effectiveness data were abstracted from patient's obstetric treatment records. Primary and secondary outcomes included:

− Participation in telehealth and in-person prenatal and postpartum appointments (primary outcomes), including:
  • Prenatal engagement: Number of prenatal visits, patient portal messages, and patient telephone calls to the obstetrics team.
  • Postpartum engagement: Receipt of any postpartum care visit with a physician, midwife, Advanced Practice Registered Nurse (APRN), community health worker/peer doula, or lactation consultant during the first 3-months postpartum.
  • The number of visits with a provider, patient portal messages, and patient telephone calls during the first three months postpartum.
− Preterm birth, defined according to the American College of Gynecology definition as birth occurring before 37 weeks' gestation (secondary outcome) (36).
− Perinatal complications (e.g., hypertensive disorders, pregestational diabetes, vaginal bleeding, fetal growth restriction, infection; Secondary outcomes).

Data were collected after participants were at least 3-months postpartum. Participants records were reviewed from their first contact with obstetrics regarding the pregnancy episode through 3-months postpartum. Data on types of care received were abstracted from clinician and RSW notes, including contacts with the obstetric team through the patient portal or phone calls. Perinatal outcomes data, including, perinatal complications which might impact gestational age at delivery (e.g., hypertensive disorders, pregestational diabetes, vaginal bleeding, fetal growth restriction, and infection), pregnancy outcomes, and infant feeding were abstracted from obstetric delivery notes and hospital discharge summaries. Preterm birth was defined as a binary variable, according to the American College of Obstetricians and Gynecologists (ACOG) definition (37).

### Analysis

#### Qualitative analysis

Qualitative interviews were transcribed and analyzed thematically, using the domains of the Consolidated Framework for Implementation Research (CFIR) as a heuristic framework to organize study data. Data were transcribed manually by AM, then transcripts were uploaded for coding to Atlas.ti (38). The first cycle coding tree was developed deductively based on the CFIR (2009) (34) domains by DG, and validated through discussions with study team members (AM, LL, KS, MA), discrepancies were resolved through team consensus. This initial coding was completed proximal to the active implementation of the cell phone program in 2021, however, in 2022 the authors of CFIR concurrently published an important update to their initial framework (31). We understood this update to be highly resonant with our work on reducing disparities in healthcare access and implementing innovations to promote engagement, as the expanded CFIR framework is better suited to explore the intersecting relationships between the process of implementing the innovation (the phone/digital access program), the outer and inner setting domains as perceived by implementers (e.g., human-equality centeredness), as well as the recipients' (patients') experience of power relationships operating within both settings which might impact their experience of the innovation. This deeper analysis is ongoing and beyond the scope of the current manuscript. Here, because the focus of this paper is on the effectiveness of the smartphone program itself, we report on implementation *outcomes*, as described by Damschroder and colleagues in the CFIR Outcomes Addendum as (1) the adoption, implementation, and sustainment of an innovation; and (2) “*the success or failure of the innovation, based on the impact of the innovation on three important constituents: innovation recipients, innovation deliverers, and key decision-makers*” (39)*.* We analyzed these data, relevant to implementation success and the impact of the smartphone program, deductively using CFIR constructs as level 1 codes (31, 40).

#### Quantitative analysis

Descriptive statistics were used to examine phone utilization outcomes. Categorical variables were summarized as frequencies. Differences between the three cohorts were compared using chi-square tests or Fisher's exact tests, when cell counts were less than 5 events. Continuous variables were summarized as means (m) and standard deviations (sd), and ranges were included for non-normally distributed data. Analysis of Variance (ANOVA) was used to examine differences in continuous variables for normally distributed data and the Kruskal–Wallis test for non-normally distributed data. We then developed multivariable logistic regression models to examine the association between cohort, preterm delivery, and postpartum visit attendance. Postpartum obstetric visit attendance was defined as attending at least one appointment during the first three months postpartum with an obstetric provider, including a physician, midwife, Advanced Practice Registered Nurse (APRN), lactation consultant, or community health worker/doula. While we initially considered prenatal care utilization as an outcome of interested, participants in the smartphone access group generally received the smartphone upon engagement in prenatal care, so we focused the regression models on postpartum care attendance, after patients had received the smartphone. Age, race and ethnicity were not included because of high standard error values caused by the homogeneity of the sample. Models were adjusted for psychiatric diagnosis, estimated gestational age at entry to prenatal care, and mode of delivery. Because estimated gestational age at entry to prenatal care was non-normally distributed, this variable was converted to an indicator variable denoting during which trimester of pregnancy patients entered prenatal care. Covariates were added to the model using a forward selection method.

The rate of missing data was examined for each variable. The study team considered complete case analysis and multiple imputation approaches. Infant disposition at discharge was the variable with the most missing data. This variable was missing for 12 (9.2%) participants, with data missing for 9.1% (*n* = 4) Cohort 1 patients, 7.0% (*n* = 3) Cohort 2 patients, and 11.4% (*n* = 5) Cohort 3 patients. With less than 10% of data missing for each variable and because data were missing completely at random, complete cases analyses were conducted (41, 42).

To examine whether this pilot project was powered to detect group differences in the rate of participants attending a postpartum care visit, we conducted a simulation-based power analysis, using Stata, Version 19 (43). We estimated the power of detecting differences in prenatal care visit attendance in the logistic regression model, with three cohorts of patients. With approximately a 20% difference in postpartum care attendance between Cohort 1 and Cohorts 2 and 3, we simulated 1,000 datasets fitting logistic regression models, setting the alpha level at 0.05. Results of the Joint Wald Test was 0.64, suggesting the study was underpowered to detect an overall difference in postpartum care.

## Results

### Aim 1: implementation outcomes and impact of the free smartphone program

#### Qualitative impact subthemes

We conducted semi-structured interviews with 8 smartphone recipients, 6 staff members involved with the smartphone program in a variety of roles, and 2 OBGYN administrators and analyzed them deductively utilizing the revised CFIR framework. All interview participants (recipients and staff) strongly endorsed the phone program, with patients highlighting having access to providers in real time through phone calls, rather than having to wait to get somewhere where internet was available; staff endorsed a sense of relief in being able to reach difficult to reach patients and the sense of doing something positive in a time when people had very limited resources or ability for self-care. All staff felt the program was working well and explained the workflows through which patients were identified as being in need. With regards to adoption, Obstetric Department administrators spoke specifically of the economic needs in their surrounding communities and the alignment with overall department goals of reducing disparities. CFIR-based themes and representative quotes are included in Table 1.

**Table 1:** Implementation and innovation outcomes for the free smartphone program (39).

Implementation Outcomes (success vs. failure)	
Adoption	“I think we are just at the very tipping point for what we need to do for patients, uh, cell phones are great but there are way bigger issues than them needing cell phones like having food on the table for their family, for them having rides to come in for their appointments, for them to have a support person to go to when they feel alone or them to have daycare. There are so many things that we need to do. I think the cell phones are great but it is really just the very tipping point of what we need to do for these patients”. (Administrative leader)
Implementation	“So far so good, its going great. I hope we can continue this forever and ever”. (OB Staff member)
Sustainment	(Demonstrated quantitatively: # of phones delivered over time and their utilization)
Impact of the Innovation	
• On Recipients	“Interviewer: And then before the prepaid phone, uh, card, was the, was the wifi texting the primary way of communicating with your provider? Participant 2: Yes Interviewer: … what is your primary way that you communicate? Participant 2: Through my phone, they call, they uh, call my phone now…And I can call them now (patient who received a phone) Interviewer: What do you think would make the program more successful? Participant 2: Honestly, I don't think there's much more you could do to make it any more than it already is”. “Interviewer: The first question is, before enrolling in the program were you able to contact your healthcare team using a phone? Participant 3: um, it was, occasionally I had one [a phone] and occasionally I didn't. So some months when I could afford it I would have one and then others when I couldn't, I wouldn't. Interviewer: Okay. What kind of contact was possible for you before this program? Participant 3: Um, internet. I would have wifi so I would use email”.
• On Deliverers	“In the two and a half years I have worked here one of the big barriers we have identified is trying to connect with patients, they either don't have a phone due to lack of finances or they have a phone but because of lack of finances are unable to keep the phone going. So in trying to connect with them via either phone or text message there is no way because their phone has been shut off or they can only make phone calls or text messages when they are near Wifi. Meaning, if they don't have internet at their home they have to either walk somewhere, which is 99.99% of the time because they don't have transportation, to a public place like McDonald's or Duncan Doughnuts in order to be able to use their phone …. we were very excited to learn that we got the grant and that we were going to be able to offer phones and phone cards to our gals because obviously in this day in age having a cell phone is pretty much, you have to have one regardless. So to be able to offer that now to these women who otherwise would have no communication especially now during COVID when so many of them are isolated, I was jumping up and down that's how happy I was”. (Community Health Worker) “Everybody [on clinic staff] is very happy that we have it [the phone program], just going back to missed appointments and secretaries or schedulers not being able to get a hold of patients and then reaching out to us to get a hold of them, so it has been positive feedback from everybody, everyone has been very happy about this”. (Registered nurse)
• On Decision-Makers	“I think we all, uh, come from different walks of life and I have definitely, you know, told [PI] my own personal story about why this particular group of patients is near and dear to my heart. But I think on that same aspect, um, some people do, you know we do have a problem and I think it is everywhere and we see this a lot, um, you know with a lot of social injustice that is happening in the world right now”. (OB Department Administrator)

#### Smartphone access program usability

**Figure 1:**
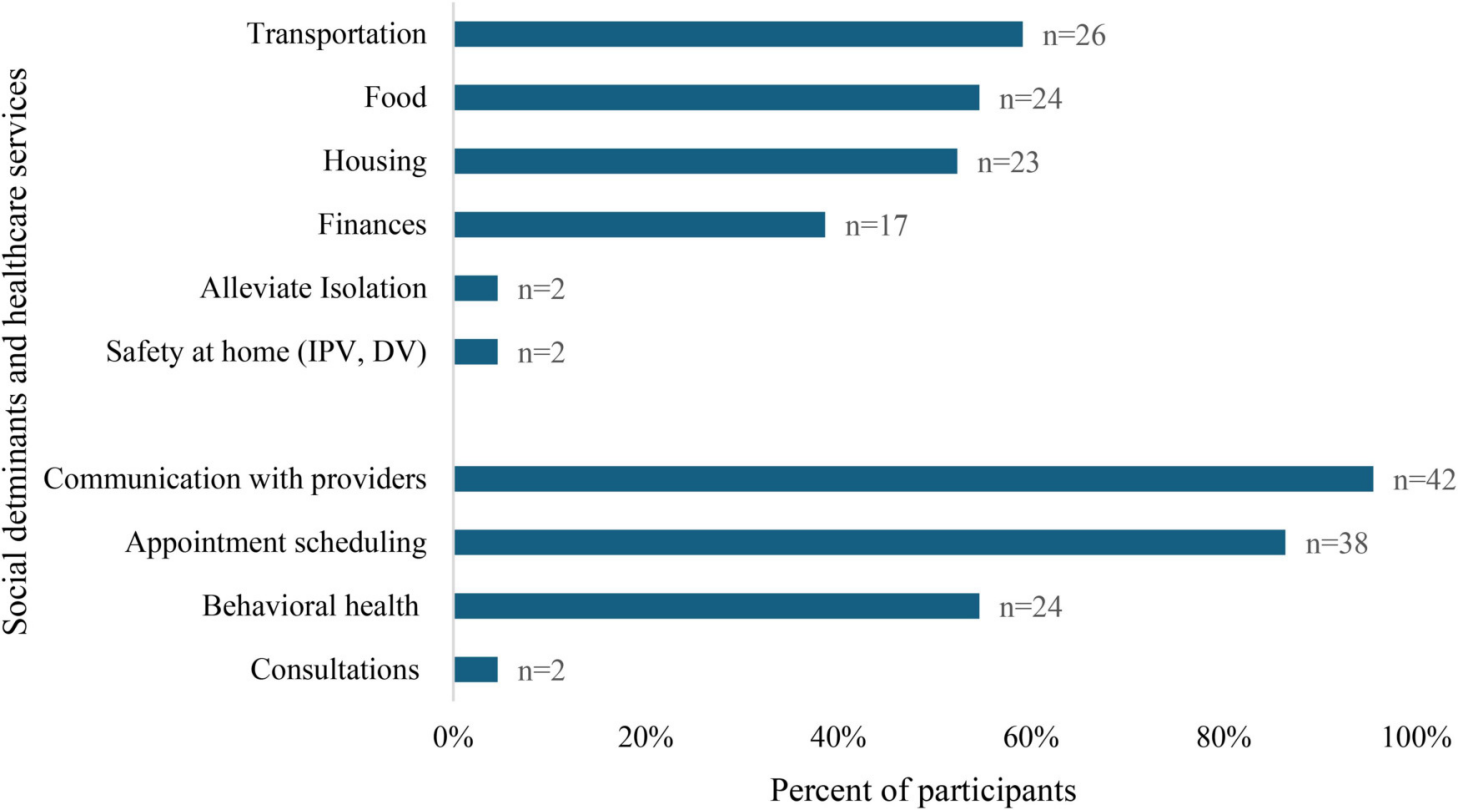
Self-reported smartphone utilization to address social determinants of health and access healthcare services by pregnant and postpartum patients enrolled in the phone access program (*n* = 44).

From 2021 to 2024, 44 obstetric patients with SUD received smartphones (Cohort 1; Table 2). Most phones were initially deployed during pregnancy (*n* = 37, 84.1%), seven patients with late entry to care received phones postpartum (*n* = 7, 16.3%). Participants attended an average of 3.7 prenatal care visits (sd = 4.0 visits) before enrolling in the phone access program, with 13.6% (*n* = 6) of participants attending no in-clinic or virtual appointments before enrolling in the program. Patients used the smartphones and data plans for an average of 162 days (range: 0–450 days). Only 5 (11.4%) required a replacement phone. Participants utilized their smartphones to access a variety of critical needs, including arranging transportation through the Medicaid-sponsored transportation or friends (59.1%), food (54.5%), and housing (52.3%), in addition to engaging in healthcare services and communicating with healthcare providers (95.5%; Figure 1). The cost of each smartphone was approximately $35, and data cards were $45–$50/month over the study period.

**Table 2:** Smartphone utilization data and participation in obstetric care for patients with SUD receiving a cell phone (*n* = 44).

Smartphone utilization and prenatal care variables	Cohort 1: Patients with SUD diagnosis receiving a cell phone (*n* = 44)
Cell Phone Utilization	
Trimester of phone receipt, *n* (%)	
First trimester (1–13 weeks)	4 (9.3%)
Second trimester (14–27 weeks)	18 (41.9%)
Third trimester (28–40 weeks)	14 (32.6%)
At delivery or postpartum	7 (16.3%)
Duration of cell phone support in days, m (sd)	162 (85) days
Range:	0–450 days
Replacement cell phone needed, *n* (%)	5 (11.4%)
Engagement in Prenatal Care Before Phone Deployment	
Total Number of prenatal care visits before phone deployment, m (sd)	3.7 (4.0)
Range:	0–15 visits
Visit type prior to cell phone deployment, *n* (%)	
In clinic	36 (81.8%)
Virtual	2 (4.6%)
None	6 (13.6%)

### Aim 2: preliminary effectiveness of the smartphone access program

#### Participant characteristics

Demographic, substance use, and psychiatric characteristics were compared across the three cohorts of patients. No significant differences in age, race, or ethnicity were found across groups (Table 3). Compared to patients in Cohort 2 (SUD, not receiving a phone) and Cohort 3 (No SUD, not receiving a phone), patients in the smartphone access program (Cohort 1) were more likely to be enrolled in Medicaid (Fisher's Exact Test [FET], *p* = 0.0001) and have a diagnosis of depression (*χ*^2^ (2) = 7.39, *p* = 0.03) or posttraumatic stress disorder (PTSD; FET, *p* = 0.05).

**Table 3:** Demographics, substance use characteristics, and psychiatric diagnoses among participants (*n* = 131).

Demographic, substance use, and psychiatric variables	Overall (*n* = 131)	Cohort 1: patients with SUD diagnosis receiving a cell phone (*n* = 44)	Cohort 2: patients with SUD diagnosis who did not receive a cell phone (*n* = 43)	Cohort 3: patients with no SUD diagnosis who did not receive a cell phone (*n* = 44)	*p* value^a^
Age years, m (sd)	29.6 (5.1)	29.7 (5.8)	28.9 (5.2)	30.2 (4.1)	0.44
Range	19–40 years	20–40 years	19–38 years	22–40 years	
Race and Ethnicity^b^, *n* (%)					
White	119 (90.8%)	37 (84.1%)	41 (95.4%)	41 (93.2%)	0.15
Black or African American	5 (3.8%)	4 (9.1%)	0 (0.0%)	1 (2.3%)	0.13
American Indian/Alaska Native	2 (1.5%)	1 (2.3%)	0 (0.0%)	1 (2.3%)	0.61
Native Hawaiian or Other Pacific Islander	2 (1.5%)	0 (0.0%)	2 (4.6%)	0 (0.0%)	0.11
Asian	1 (0.8%)	0 (0.0%)	0 (0.0%)	1 (2.3%)	0.37
Unknown or Not Reported	2 (1.5%)	2 (4.6%)	0 (0.0%)	0 (0.0%)	0.33
Latina/Latino/Latinx	2 (1.5%)	1 (2.3%)	0 (0.0%)	1 (2.3%)	0.6
Payor, *n* (%)					<0.001
Medicaid	89 (67.9%)	43 (97.7%)	36 (83.7%)	10 (11.2%)	
Commercial Insurance	37 (28.2%)	0 (0.0%)	4 (9.3%)	33 (89.2%)	
Military Insurance	1 (0.8%)	0 (0.0%)	0 (0.0%)	1 (2.3%)	
Uninsured	2 (1.5%)	0 (0.0%)	2 (4.7%)	0 (0.0%)	
Medicare	1 (0.8%)	0 (0.0%)	1 (2.3%)	0 (0.0%)	
Other	1 (0.8%)	1 (2.3%)	0 (0.0%)	0 (0.0%)	
Rural Code, *n* (%)					0.23
1–25 miles	74 (56.5%)	22 (50.0%)	25 (58.1%)	27 (61.4%)	
26–50 miles	37 (28.2%)	14 (31.8%)	13 (30.2%)	10 (22.7%)	
51–75 miles	14 (10.7%)	3 (6.8%)	4 (9.3%)	7 (15.9%)	
76–10 miles	4 (3.1%)	3 (6.8%)	1 (2.3%)	0 (0.0%)	
>100 miles	2 (1.5%)	2 (4.6%)	0 (0.0%)	0 (0.0%)	
Parity, median	3	3	3	2	<0.05
Range	1–11	1–11	1–7	1–9	
Gestational Age at Entry to Prenatal Care, median	9	13	9	8.5	<0.04
Range	5–39	5–39	5–37	6–37	
English language preference, *n* (%)	131 (100%)	44 (100%)	43 (100%)	44 (100%)	0.98
Substance use diagnosis, *n* (%)					
Alcohol	10 (7.6%)	3 (6.8%)	7 (16.3%)	0 (0.0%)	<0.02
Opioid	65 (49.6%)	40 (90.9%)	24 (55.8%)	0 (0.0%)	<0.001
Stimulant	15 (11.5%)	10 (22.7%)	5 (11.6%)	0 (0.0%)	<0.01
Cocaine	8 (6.1%)	6 (13.6%)	2 (4.7%)	0 (0.0%)	<0.05
Cannabis	43 (32.8%)	26 (59.1%)	16 (37.2%)	0 (0.0%)	<0.001
Tobacco	44 (33.6%)	27 (61.4%)	17 (39.5%)	0 (0.0%)	<0.001
Other	7 (5.3%)	4 (9.1%)	3 (6.9%)	0 (0.0%)	0.13
Psychiatric diagnosis, *n* (%)	100 (76.3%)	38 (86.4%)	38 (88.4%)	24 (54.6%)	<0.001
Anxiety	64 (48.9%)	22 (50.0%)	21 (48.8%)	21 (47.7%)	0.98
Depression	72 (54.9%)	29 (65.9%)	26 (60.5%)	17 (38.6%)	<0.05
Bipolar disorder	8 (6.1%)	3 (6.8%)	4 (9.3%)	1 (2.3%)	0.36
PTSD	21 (16.0%)	12 (27.3%)	4 (9.3%)	5 (11.4%)	0.05
Anorexia	0 (0.0%)	0 (0.0%)	0 (0.0%)	0 (0.0%)	--
Bulimia	0 (0.0%)	0 (0.0%)	0 (0.0%)	0 (0.0%)	--
Other	18 (13.7%)	8 (18.2%)	8 (18.6%)	2 (4.6%)	0.07

^a^ Normal continuous variables compared using ANOVA, Non-normal variables (parity and gestational age at entry to prenatal care) compared with Kruskal–Wallis test; Categorical variables compared using chi-square test.

^b^ Some participants belonged to more than one racial or ethnic group.

#### Prenatal care utilization

In comparison to patients in Cohorts 2 and 3, patients in Cohort 1 attended fewer prenatal visits (H(2) = 15.8, *p* = 0.0004) and were less likely to send patient portal messages (FET, *p* = 0.001) or have telephone calls (FET, *p* = 0.001) with the obstetrics team (Table 4). Cohort 1 also entered prenatal care at a significantly later gestational age (13 weeks [Cohort 1] vs. 9 weeks [Cohort 2] vs. 8.5 weeks [Cohort 3]; H(2) = 6.9, *p* = 0.03).

**Table 4:** Comparison of prenatal care participation, delivery outcomes, and postpartum care participation by cohort (*n* = 131) using chi-square and kruskal–wallis tests.

Prenatal and postpartum variables	Overall (*n* = 131)	Cohort 1: patients with SUD diagnosis receiving a smartphone (*n* = 44)	Cohort 2: patients with SUD diagnosis who did not receive a smartphone (*n* = 43)	Cohort 3: patients with no SUD diagnosis who did not receive a smartphone (*n* = 44)	*p* value
Number of prenatal messages, telephone calls, and provider visits					
Prenatal visits, m (sd)	9.2 (4.2)	7.0 (4.7)	10.0 (3.2)	10.7 (3.5)	<0.001
Range:	0–15	0–15	2–15	2–15	
Patient portal messages, *n* (%)					<0.01
0 messages	26 (19.9%)	19 (43.2%)	4 (9.3%)	3 (6.8%)	
1–5 messages	34 (25.9%)	9 (20.5%)	10 (23.3%)	15 (32.1%)	
6–10 messages	38 (28.2%)	9 (20.5%)	16 (37.2%)	12 (27.3%)	
10+ messages	34 (25.9%)	7 (15.9%)	13 (30.2%)	14 (31.8%)	
Telephone calls, *n* (%)					<0.01
0 calls	10 (7.7%)	7 (15.9%)	1 (2.3%)	2 (4.7%)	
1–5 calls	44 (33.9%)	8 (19.2%)	13 (30.2%)	23 (53.5%)	
6–10 calls	42 (32.3%)	14 (31.8%)	14 (32.6%)	14 (32.6%)	
11+ calls	34 (26.2%)	15 (34.1%)	15 (34.9%)	4 (9.3%)	
Perinatal complications and delivery outcomes					
Mode of delivery^a^, *n* (%)					0.77
Spontaneous vaginal	73 (57.5%)	27 (62.8%)	24 (57.1%)	22 (52.4%)	
Operative vaginal	13 (11.0%)	3 (7.0%)	6 (14.3%)	5 (11.9%)	
Cesarean section	40 (31.5%)	13 (30.2%)	12 (28.6%)	15 (35.7%)	
Perinatal Complications, *n* (%)					
Hypertensive disorders	12 (9.2%)	2 (4.6%)	5 (11.6%)	5 (11.4%)	0.45
Diabetes (pre-gestational/gestation)	4 (3.1%)	0 (0.0%)	1 (2.3%)	3 (6.8%)	0.22
Vaginal bleeding during pregnancy	1 (0.8%)	0 (0.0%)	0 (0.0%)	1 (2.3%)	0.37
Fetal growth restriction	5 (3.8%)	3 (6.8%)	1 (2.3%)	1 (2.3%)	0.62
Infection	2 (1.5%)	2 (4.6%)	0 (0.0%)	0 (0.0%)	0.33
Preterm labor	29 (22.1%)	13 (29.6%)	12 (27.9%)	4 (9.1%)	<0.05
Infant birthweight grams, m (sd)	3,121 (630)	3,013 (663)	3,132 (607)	3,212 (624)	0.38
Infant feeding initiated, *n* (%)^b^					
Direct breastfeeding	84 (64.1%)	18 (40.9%)	30 (69.8%)	36 (81.8%)	<0.001
Pumped breastmilk	37 (28.2%)	21 (47.7%)	11 (25.6%)	5 (11.4%)	<0.01
PDHM	5 (3.8%)	3 (6.8%)	1 (2.3%)	1 (2.3%)	0.62
Formula	21 (16.0%)	14 (31.8%)	5 (11.6%)	2 (4.6%)	<0.01
Unknown	15 (11.5%)	9 (20.5%)	5 (11.6%)	1 (2.3%)	<0.05
Infant disposition at discharge, *n* (%)^a^					<0.001
Home with parent	108 (90.8%)	29 (72.5%)	40 (100%)	39 (100%)	
CPS custody/placement	8 (6.7%)	8 (20.0%)	0.00%	0.00%	
Home with other family member	3 (2.5%)	3 (7.5%)	0.00%	0.00%	
Number of postpartum messages, telephone calls, and provider visits					
Postpartum care visit, *n* (%)	115 (87.8%)	36 (81.8%)	39 (90.7%)	40 (90.9%)	0.33
Postpartum provider visits, m (sd)	1.4 (1.2)	1.2 (1.2)	1.6 (1.1)	1.7 (1.3)	0.08
Range	0–6	0–4	0–5	0–6	
Patient portal messages, *n* (%)					<0.01
0 messages	46 (35.1%)	27 (61.4%)	10 (23.3%)	9 (20.5%)	
1–2 messages	30 (22.9%)	4 (9.1%)	12 (27.9%)	14 (31.8%)	
3–4 messages	27 (20.6%)	5 (11.4%)	13 (30.2%)	9 (20.5%)	
5–13 messages	28 (21.4%)	8 (18.2%)	8 (18.6%)	12 (27.3%)	
Telephone calls, *n* (%)					0.11
0 messages	33 (25.2%)	9 (20.5%)	8 (18.6%)	16 (36.4%)	
1–2 calls	50 (38.2%)	14 (31.8%)	17 (39.5%)	19 (43.2%)	
3–4 calls	26 (19.9%)	13 (29.6%)	8 (18.6%)	5 (11.4%)	
5+ calls	22 (16.8%)	8 (18.2%)	10 (23.3%)	4 (9.1%)	
Postpartum engagement type, *n* (%)					
CHW/doula	39 (29.8%)	29 (65.9%)	10 (23.3%)	0 (0.0%)	<0.001
Recovery support worker	24 (18.3%)	17 (38.6%)	7 (16.3%)	0 (0.0%)	<0.001
Midwife	75 (57.3%)	21 (47.7%)	26 (60.5%)	28 (63.6%)	0.28
Physician	34 (25.9%)	11 (25.0%)	11 (25.6%)	12 (27.3%)	0.97
Behavioral Health	29 (22.1%)	16 (36.4%)	11 (25.6%)	2 (4.6%)	<0.01
Lactation Consultant	24 (18.3%)	5 (11.4%)	8 (18.6%)	11 (25.0%)	0.25
MAT/MOUD	8 (6.1%)	5 (11.4%)	3 (7.0%)	0 (0.0%)	0.08
None	12 (9.2%)	5 (11.4%)	4 (9.3%)	3 (6.8%)	0.81

^a^ Mode of delivery missing for 4 (3.1%) participants; Infant disposition at discharge missing for 12 participants.

^b^ Participants could engage in more than 1 type of infant feeding.

#### Delivery outcomes

Patients with SUD (Cohorts 1 [29.6%] and 2 [27.9%)]) were significantly more likely to experience preterm labor compared to patients without SUD (Cohort 3) (29.6%, 27.9%, and 9.1% respectively; FET, *p* = 0.03; Table 4). Controlling for estimated age at entry to prenatal care, psychiatric diagnosis, and mode of delivery, patients in Cohort 1 (Odds Ratio [OR] = 4.25, 95% Confidence Intervals [CI] = 1.11, 16.4) and Cohort 2 (OR = 4.10, 95% CI = 1.09, 15.4) were at significantly increased risk for preterm delivery (Table 5).

**Table 5:** Univariate and multivariable association between treatment cohort and the number of risk for preterm delivery and prenatal care engagement.

Treatment cohort and covariates	Preterm delivery		Prenatal care engagement	
	Logistic regression model		Logistic regression model	
	Univariate odds ratio (95% CI)	Multivariable odds ratio (95% CI)^a^	Univariate odds ratio (95% CI)	Multivariable odds ratio (95% CI)^a^
Treatment cohort				
Cohort 1 (SUD diagnosis + smartphone)	4.19 (1.24, 14.1)	4.25 (1.11, 16.4)	0.45 (0.12, 1.62)	0.52 (0.11, 2.56)
Cohort 2 (SUD diagnosis, no smartphone)	3.87 (1.14, 13.2)	4.10 (1.09, 15.4)	0.98 (0.23, 4.17)	1.19 (0.20, 7.12)
Cohort 3 (No SUD, no smartphone)	1.00 (reference)	1.00 (reference)	1.00 (reference)	1.00 (reference)
Gestational age at entry to prenatal care				
First trimester	1.00 (reference)	1.00 (reference)	1.00 (reference)	1.00 (reference)
Second trimester	2.59 (1.03, 6.48)	1.84 (0.68, 4.96)	0.33 (0.10, 1.07)	0.34 (0.09, 1.27)
Third trimester	0.42 (0.05, 3.53)	0.35 (0.04, 3.06)	0.38 (0.07, 2.14)	0.79 (0.08, 7.78)
Psychiatric diagnosis				
No diagnosis	1.00 (reference)	1.00 (reference)	1.00 (reference)	1.00 (reference)
One or more diagnoses	1.24 (0.46, 3.40)	0.76 (0.24, 2.44)	0.72 (0.19, 2.70)	1.13 (0.25, 5.03)
Mode of delivery				
Spontaneous vaginal	1.00 (reference)	1.00 (reference)	1.00 (reference)	1.00 (reference)
Operative vaginal	0.59 (0.12, 2.93)	0.67 (0.13, 3.56)	1.83 (0.21, 15.7)	1.02 (0.11, 9.71)
Cesarean section	1.35 (0.56, 3.29)	1.52 (0.59, 3.91)	1.27 (0.36, 4.40)	1.05 (0.28, 3.91)

^a^ Adjusted for all covariates in table.

Patients in Cohort 1 were also less likely to initiate direct breastfeeding (*χ*^2^(2) = 16.9, *p* = 0.000) or feeding with pumped breastmilk (*χ*^2^(2) = 14.6, *p* = 0.001) and were more likely to provide formula (FET, *p* = 0.002). Finally, infant disposition at hospital discharge differed significantly across cohorts. While all infants in Cohort 2 and 3 were discharged in custody of their birth parent, 20.0% (*n* = 8) of infants in Cohort 1 were discharged into child protective services (CPS) custody and 7.5% (*n* = 3) were discharged with another family member (FET, *p* = 0.000).

#### Postpartum care utilization

In contrast to the marked differences in prenatal care engagement, psychiatric comorbidities, substance use and infant outcomes for Cohort 1, there was no overall difference in the number of postpartum provider visits by cohort (Table 4; H(2) = 5.1, *p* = 0.08) or the proportion of patients attending at least one postpartum obstetric visit (*χ*^2^(2) = 2.20, *p* = 0.33). Adjusting for estimated age at entry to prenatal care, psychiatric diagnosis, and mode of delivery, patients in Cohort 1 (OR = 0.52, 95% CI = 0.11, 2.56) and Cohort 2 (OR = 1.19, 95% CI = 0.20, 7.12) did not have significantly difference rates of postpartum visit attendance compared to patients in Cohort 1 (Table 5). Notably, there were no significant differences in the rate of postpartum engagement with a midwife (*χ*^2^(2) = 2.54, *p* = 0.28), OB/GYN physician (*χ*^2^(2) = 0.06, *p* = 0.97), or lactation consultant (*χ*^2^(2) = 2.74, *p* = 0.25) by cohort. Patients with SUD (Cohorts 1 and 2) both had high rates of engagement with recovery support and behavioral health services postpartum (Table 4). Most significantly, more than half of patients in Cohort 1 received postpartum Community Health Worker (CHW)/doula support (65.9%), and more than a third (38.6%) received recovery support.

## Discussion

This pilot cohort study examined the implementation of a smartphone access program for pregnant and perinatal patients with SUD, its utilization by patients, and explored the effectiveness of this simple intervention on promoting participation in prenatal and postpartum care services for patients with very limited resources and poor attendance history. Initial findings support the implementability of a smartphone access program into a rural obstetric setting. During the first three years of the program, 44 patients who initially lacked access to a phone receiving and using smartphones and monthly data plans were given access to smartphones and data plans with minimal interruptions in service during pregnancy and postpartum. Patients with the greatest economic and social vulnerability who received a phone subsequently had high levels of engagement with their healthcare teams in the postpartum period, decreasing the disparity in postpartum engagement between this high risk group and other postpartum patients at the same institution.

Notably, interviews with patients, providers, and OB staff universally supported the positive impact of the program, with all participants noting that the program was easily adopted into the OB setting. All interviewees highlighted how access to a smartphone and data plan allowed patients, providers, and staff to more easily communicate.

**Figure 2:**
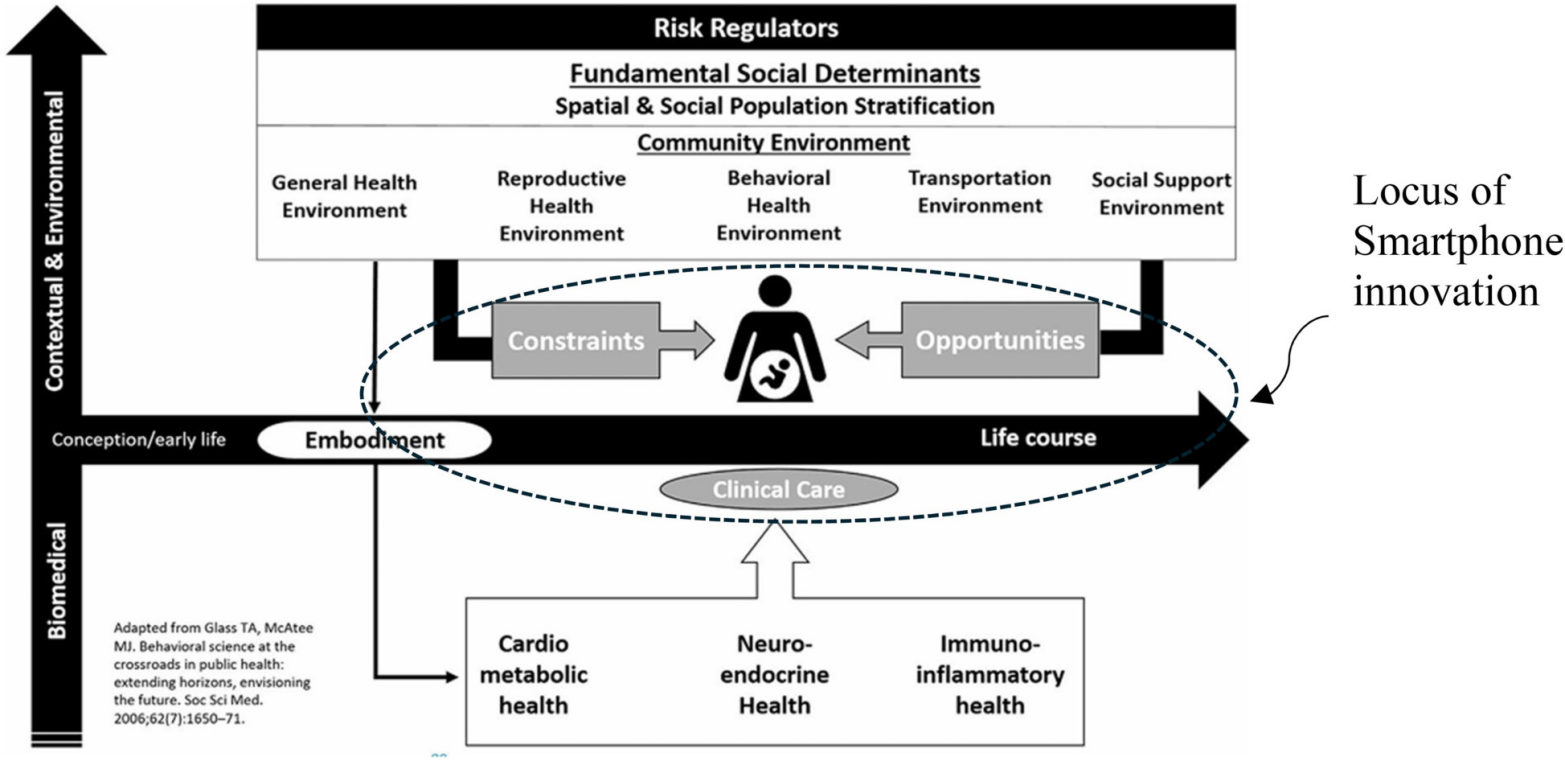
Facilitation of opportunity within the health equity framework for maternal Mortality (15).

As illustrated by the Kramer health equity framework for maternal mortality (15), a woman's health at the time she becomes pregnant is an embodiment of her cumulative life experiences in combination with biomedical contributors; which interacts with the environment in which she moves during pregnancy and is mitigated (or exacerbated) by the clinical care she receives. This environment itself presents both opportunities for risk reduction and constraints which increase her risk of morbidity and mortality. It is here that the smartphone and the connections it provides (to care, resources, and supports) achieve their impact- transforming a constrained environment to one of opportunity (Figure 2).

Maternal mortality data regionally and nationally demonstrates a strong association between mortality, active substance use, other mental health conditions, Medicaid insurance, and social stressors (1–8, 44). Our findings reveal that the group of patients with SUD who could not afford a cell phone (Cohort 1) were also more likely to have co-occurring psychiatric conditions and active drug use during pregnancy and were more likely to be insured by Medicaid than their more financially stable peers with SUD (Cohort 2) and pregnant patients generally (Cohort 3). This more socially vulnerable group engaged later in prenatal care, received fewer prenatal visits than patients with SUD who did not need a phone, and were more likely to experience negative perinatal outcomes (e.g., preterm labor, loss of child custody). A significant body of research suggests that addressing SDOH during pregnancy is crucial to supporting engagement in healthcare services and improving outcomes for patients and families (45, 46), our program participants used their smartphones to access multiple social supports, including food, housing, and transportation as well as medical care.

Our findings show that disparities in access at the onset of prenatal care may be reversed by interventions in the obstetric settings, as is evident in the equal levels of postpartum engagement among patients in the smartphone program compared to obstetric patients more generally. Additionally, the robust engagement in mental health care and recovery support as well as encounters with obstetric providers seen in Cohort 1 are markedly different from the limited prenatal engagement seen in the same group. Together, these findings suggest that a simple intervention- access to a smartphone, a data plan, and support to operationalize them- can materially change patients' relationship with and ability to participate in postpartum and behavioral health care and facilitate access the community-level supports critically important in improving outcomes. This is encouraging, given the increased risk of fatal overdose seen in the postpartum period for patients with SUD.

Although a few patients enrolled in the smartphone access program at the onset of participation in prenatal care, most attended several prenatal care sessions prior to enrollment, and others received minimal prenatal care and enrolled at delivery. Considering strategies to provide smartphone access earlier during pregnancy could facilitate earlier enrollment in prenatal care for patients with multiple social vulnerabilities. These strategies might include coordinating with local recovery and harm reduction programs to encourage enrollment in the smartphone program early in pregnancy, and creating access points for the program, such as a helpline, outside of the traditional obstetric setting. Our team includes people with lived experience whose contribution to designing the smartphone program enhanced its acceptability and effectiveness, consistent with other studies of co-design for vulnerable populations (47–50).

Our smartphone access program was operationalized by RSWs and Community Health Workers who provide navigation support to patients beyond traditional obstetric care services, another factor which may have improved trust and engagement in care (51–55). It is important to note that access to the RSW was primarily by phone/text, and our innovative model includes both the phone and peer facilitation. This is aligned with other studies which note that pairing a trained healthcare worker with a phone may synergistically increase engagement in healthcare (27, 56). For example, pregnant women in Nigeria who received mobiles phone described free and easy communication with a health support worker as the most important benefit of having the phone (28). Future studies should therefore consider how engaging recovery support workers, peer doulas, or community health workers could amplify the impacts of providing smartphone access for pregnant and postpartum people with SUD.

### Limitations

This small, retrospective cohort study was conducted at a single academic medical center in a rural region. Results may not be generalizable to more urban settings, different practice types, and culturally and linguistically diverse populations. Future work should include community engaged participatory research with a range of communities to adapt and disseminate this innovation.

With only 131 participants, this study was underpowered to detect a 20% difference in rates of postpartum care attendance. Therefore, the lack of a statistically significant difference in postpartum care between cohorts in the logistic regression model was likely due to the small study sample size. Additionally, we did not apply a multiple testing correction, such as a Holm correction, on univariate tests because this pilot study was exploratory and underpowered to detect between group differences. The findings should not solely be interpreted based on the *p*-values or strict hypothesis testing. However, from the clinical perspective, a non-difference between Cohort 1 and Cohorts 2 and 3 represents a meaningful success as postpartum care is protective against perinatal morbidity and mortality, especially in the context of SUD (57). A larger, multisite study is currently being planned to examine factors contributing to the smartphone program feasibility and acceptability, using the revised Consolidated Framework for Implementation Research (CFIR) (31) as a framework to more systematically examine program implementation at a second site. Finally, the current study was unable to explore the sustainability of the free smartphone program.

Rural communities face unique challenges to accessing obstetric care, including lengthy travel to appointments, financial strain and inadequate digital access to care with poor broadband and device accessibility (58, 59). For rural pregnant and postpartum patients with SUD, virtual connection with healthcare providers can be a critical lifeline to care, yet digital disparities also widen barriers to care in an era where digital access is expected. Numerous barriers prevent pregnant and postpartum people with SUD from accessing healthcare services, including the cost of maintaining a phone, and the presence of other digital disparities such as access to broadband services. These challenges potentiate structural barriers such as isolation and poverty already experienced by marginalized rural patients, resulting in increased risk of perinatal complications. Results from this small pilot study suggest that implementing a free smartphone program is possible in a rural obstetric setting. In this study, smartphones were highly utilized by patients to engage in perinatal care, access social services, and participate in behavioral health and recovery support, thus reducing risk factors associated with perinatal mortality. Maternity care programs should be aware of and address digital disparities as a critical component of equal access for all patients, including during the postpartum period, and policymakers should consider access to a smartphone as an essential component of health equity.

## Data Availability

Requests to access the datasets presented in this article should be directed to daisy.j.goodman@hitchcock.org.
